# Intra-neuronal alpha-synuclein deposition is related to cardiac noradrenergic deficiency and olfactory dysfunction in neurogenic orthostatic hypotension

**DOI:** 10.21203/rs.3.rs-3988235/v1

**Published:** 2024-03-01

**Authors:** Risa Isonaka, Patti Sullivan, Courtney Holmes, David S. Goldstein

**Affiliations:** 1 Autonomic Medicine Section, Clinical Neurosciences Program, Division of Intramural Research, National Institute of Neurological Disorders and Stroke, National Institutes of Health, Bethesda, MD

**Keywords:** synuclein, fluorodopamine, olfactory, orthostatic hypotension, Lewy

## Abstract

**Purpose:**

Neurogenic orthostatic hypotension (nOH) results from deficient reflexive delivery of norepinephrine to cardiovascular receptors in response to decreased cardiac venous return. Lewy body (LB) forms of nOH entail low ^18^F-dopamine-derived radioactivity (a measure of cardiac noradrenergic deficiency), olfactory dysfunction by the University of Pennsylvania Smell Identification Test (UPSIT), and increased deposition of alpha-synuclein (ɑ-syn) in dermal sympathetic noradrenergic nerves by the ɑ-syn-tyrosine hydroxylase (TH) colocalization index. This observational, cross-sectional study explored whether combinations of these biomarkers specifically identify LB forms of nOH.

**Methods:**

Clinical laboratory data were reviewed from patients referred for evaluation at the National Institutes of Health for chronic autonomic failure between 2011 and 2023. The cutoff value for low myocardial ^18^F-dopamine-derived radioactivity was 6,000 nCi-kg/cc-mCi, for olfactory dysfunction an UPSIT score ≤ 28, and for an increased ɑ-syn-TH colocalization index ≥ 1.57.

**Results:**

A total of 44 patients (31 LB, 13 non-LB nOH) had data for all 3 biomarkers. Compared to the non-LB group, the LB nOH group had low myocardial ^18^F-dopamine-derived radioactivity, low UPSIT scores, and high ɑ-syn-TH colocalization indexes (p<0.0001 each). Combining the 3 biomarkers completely separated the groups. Cluster analysis identified 2 distinct groups (p<0.0001) independently of the clinical diagnosis, 1 cluster corresponding exactly to LB nOH.

**Conclusion:**

LB forms of nOH feature cardiac noradrenergic deficiency, olfactory dysfunction, and increased ɑ-syn-TH colocalization in skin biopsies. Combining the data for these variables efficiently separates LB from non-LB nOH. Independently of the clinical diagnosis, this biomarker triad identifies a pathophysiologically distinct cluster of nOH patients.

## INTRODUCTION

Orthostatic hypotension is considered to be neurogenic (nOH) when the patient has persistent, consistent OH, there is no identified secondary cause, and there is evidence of decreased ability to maintain blood pressure reflexively in response to decreased venous return to the heart [11]. nOH occurs in a substantial minority of patients with Parkinson’s disease (PD) [56], most patients with multiple system atrophy (MSA), and all patients with pure autonomic failure (PAF). These diseases are synucleinopathies, involving intra-cytoplasmic deposition of the protein alpha-synuclein (α-syn). In Lewy body (LB) forms of synucleinopathy the protein typically is deposited in neurons, whereas in MSA the deposits are in glial cytoplasmic inclusions [57]. Clinically diagnosed PAF can phenoconvert to PD, MSA, or dementia with Lewy bodies (DLB) [36].

It is difficult to distinguish LB from non-LB forms of nOH by clinical examination alone. There has long been interest in identifying biomarkers that might make this distinction. The first such biomarker to be described was cardiac sympathetic neuroimaging by ^18^F-dopamine positron emission tomography (PET) [19]. ^18^F-Dopamine is especially powerful for separating PD+OH from the parkinsonian form of MSA, as virtually all patients with PD+OH have severely decreased ^18^F-dopamine-derived radioactivity, whereas most patients with MSA have normal radioactivity [43].

Patients with LB forms of nOH often have olfactory dysfunction, based on scores on the University of Pennsylvania Smell Identification Test (UPSIT), whereas in MSA olfaction usually is normal or only mildly or moderately decreased [12, 27]. Moreover, across synucleinopathy patients olfactory dysfunction is related to neuroimaging evidence of cardiac noradrenergic deficiency, both by ^18^F-dopamine PET [18] and ^123^I-metaiodobenzylguanidine (^123^I-MIBG) single photon emission computed tomography (SPECT) [41].

Neither cardiac sympathetic neuroimaging nor olfactory testing directly identifies synucleinopathy. Until relatively recently, confirming the occurrence of a LB disease required post-mortem neurohistopathology. Over about the past decade, however, evidence has accrued for increased deposition of native or S129 phosphorylated a-syn in sympathetic noradrenergic nerve fibers in skin biopsies in PD, PAF, and DLB and not in MSA [4, 6, 32]. For separating LB from non-LB forms of nOH, quantification of an α-syn-tyrosine hydroxylase (TH) colocalization index has been validated by post-mortem analyses of sympathetic ganglion tissue [32].

Previous studies have not included concurrent measurements of cardiac sympathetic innervation, olfactory function, and α-syn-TH colocalization in patients with nOH. In this study we asked whether these biomarkers in combination can distinguish LB from non-LB forms of nOH. We also addressed the converse—whether the biomarker phenotypic pattern identifies two pathophysiologically distinct groups of nOH regardless of the clinical diagnosis.

Finally, although long-term trends in cardiac sympathetic innervation have been described [40], those in olfactory function and α-syn-TH colocalization have not. We therefore also analyzed longitudinal follow-up data for these measures from subgroups of study participants who underwent serial evauations at the National Institutes of Health (NIH) Clinical Center.

## METHODS

### Study Subjects

All the participants in this observational, cross-sectional study gave written informed consent before any research procedures were done. The protocols were approved by the Institutional Review Board (IRB) of the National Institute of Neurological Disorders and Stroke (NINDS) or the IRB of the NIH. All the patients had been referred for evaluation by the Autonomic Medicine Section (AMS, formerly Clinical Neurocardiology Section) of the Division of Intramural Research of the NINDS at the NIH Clinical Center. Clinical laboratory data were reviewed from all patients referred for evaluation by the AMS at the NIH between 2011 and 2023.

### Neurogenic orthostatic hypotension

The presence or absence of nOH was determined based on beat-to-beat blood pressure responses to the Valsalva maneuver or orthostatic fractional increments in plasma norepinephrine levels [23, 29].

PAF, PD+OH, MSA, and other forms of nOH—autoimmune autonomic ganglionopathy (AAG), autoimmunity-associated autonomic failure with sympathetic denervation, AAD) were diagnosed based on previously published consensus statements [11, 14, 15, 35], case reports [20, 22, 24], and post-mortem data when available [33, 34]. We also used the UK Brain Bank criteria for PD [30], with the following exception. According to the UK Brain Bank criteria, early, prominent autonomic involvement excludes a diagnosis of PD. Findings by our group [16, 22, 25] and others [47] that OH can occur in preclinical PD question this statement.

### ^18^F-Dopamine positron emission tomography

^18^F-dopamine PET was carried out as described previously [18]. Briefly, 1 mCi of the tracer was injected intravenously over 3 minutes. The radioactivity concentration in the interventricular septum was averaged in the 5-minute dynamic frame beginning about 5 minutes after initiation of the injection (midpoint about 8 minutes). The decay-corrected radioactivity concentration, in nCi/cc, was adjusted for the administered dose per kg body mass and expressed in units of nCi-kg/cc-mCi. A cutoff value of 6,000 nCi-kg/cc-mCi was used to define low ^18^F-dopamine-derived radioactivity [25].

### University of Pennsylvania Smell Identification Test

The 40-item UPSIT was administered according to instructions [8]. The raw score was not adjusted for age or sex. A cutoff value of 28 was chosen, corresponding to moderately severe olfactory dysfunction.

### Skin biopsies for immunofluorescence confocal microscopy

The location of the skin biopsies was the C2 region of the nape of the neck. Three-mm diameter skin punch biopsy samples were placed in Zamboni fixative solution and kept at 4 °C for 18–20 hours, washed with Sorenson’s phosphate buffer (133 mM, pH 7.6), and placed in 20% glycerol for cryoprotection. Samples were embedded in optimum cutting temperature compound, frozen, sliced into 8–10 μm thick sections (Histoserv, Germantown, MD), and kept frozen at −80 °C until thawed for assay.

The primary antibodies used were as follows: rabbit anti-tyrosine hydroxylase (TH) (1:1000; Pel-Freez Biologicals, Rogers, AR), mouse IgG1 monoclonal anti-α-synuclein (α-syn) (1:1000; Santa Cruz Biotechnology, Santa Cruz, CA), and mouse IgG2_a_ monoclonal anti-alpha-smooth muscle actin (SMA) (1:400; Santa Cruz Biotechnology). For the detection of S129 phosphorylated α-syn, a recombinant monoclonal rabbit antibody was used (Abcam Inc., Waltham, MA). When this antibody was employed, the primary antibody to TH was switched to chicken anti-TH (1:500; Abcam Inc.). The primary immunoreactions were visualized with the following secondary antibodies: Alexa 488-conjugated anti-mouse IgG1 for α-syn, Alexa 555 conjugated anti-rabbit for TH, and when detecting phosphorylated α-syn Alexa 488-conjugated anti-rabbit. For chicken anti-TH, Alexa 555-conjugated anti-chicken was utilized. Additionally, Alexa 647-conjugated anti-mouse IgG2_a_ was used for SMA (all from Thermo Scientific, Inc, Rockford, IL).

### Follow-up data

Although most of this study was cross-sectional, there was a longitudinal aspect. Subgroups of the study participants had serial data for cardiac ^18^F-dopamine-derived radioactivity, UPSIT scores, and α-syn-TH colocalization indexes during follow-up evaluations at the NIH Clinical Center.

### Avoidance of biases

The confocal microscopic imaging and subsequent image analyses were conducted at the NIH by personnel who were strictly blinded as to the diagnostic group until the data were tabulated. The clinical team was also blinded as to the imaging data until the data were tabulated. Personnel analyzing PET images were blinded as to both the clinical laboratory results and skin biopsy data.

### Data Analysis and Statistics

α-Syn-TH colocalization in entire images was analyzed using Fiji software, with background subtraction as described previously [32]. α-Syn-TH colocalization indexes were calculated in the following steps: (1) normalized mean deviation product (nMDP) values from - 1.0 to +1.0 were tabulated; (2) counts corresponding to nMDP values from 0.3 to 1.0 were summed; (3) 0.1 was added, so that the sum of the counts was greater than zero; and (4) the log of the number from step (3) was calculated.

GraphPad Prism 9 for Mac (GraphPad Software, Boston, MA) was used for most of the statistical analyses and graphics. Mean values in the LB and Non-LB nOH groups were compared by independent-means t tests. XY 3-D scatterplots were created to display individual values for the 3 biomarkers and for values across years of follow-up.

Fisher’s exact test was used to compare the frequencies of abnormal biomarkers, alone or in combination, in the LB nOH and Non-LB nOH groups.

Pearson correlation coefficients were calculated for phosphorylated a-syn-TH colocalization indexes vs. native α-syn-TH colocalization indexes.

We performed a cluster analysis on the data for the 3 biomarkers using the k-means algorithm, as implemented in the KMeans function from the sklearn library in Python 3.12.1 (https://scikit-learn.org/). There were three general reasons for using KMeans. First, unlike simple correlation matrices, clustering algorithms like KMeans can discern and adapt to non-linear relationships and dependencies in the data, offering resilience against outliers and complex dynamics. Second, while multicollinearity can confound coefficient estimates in regression models, KMeans remains unaffected. This attribute stems from the algorithm’s focus on the spatial structure of the data rather than the interdependencies among variables. Third, physiological processes often operate in discrete states of equilibrium. Correlations that are apparent within individual states may not extend across states. KMeans clustering effectively delineates these states, revealing distinct patterns and guiding in-depth analysis into the underlying physiological phenomena.

The KMeans algorithm partitions the dataset into a user-defined number of clusters. Unlike methods that deduce the optimal number of clusters through intrinsic dataset properties, KMeans necessitates a pre-specified cluster count. For our analysis, we selected a bifurcation approach, hypothesizing the data could be categorically divided into 2 distinct clusters. The assignment of data points to clusters is achieved by minimizing the total squared Euclidean distance between each point and the centroid of its assigned cluster. This iterative optimization process refines the centroids and the cluster memberships until the most compact and distinct grouping is attained. Given the disparate scales of variables in our dataset, standardization is pivotal. By standardizing, we ensured that each feature contributed equally to the analysis, akin to calibrating axes in a graph for uniform scale and comparability. This structured approach to clustering facilitated a nuanced exploration of the data, uncovering patterns and relationships that might elude traditional analytical methods.

To evaluate the probability of the observed clustering occurring by chance, we considered K successes in n draws, without replacement, from a finite population of N that contains exactly K objects with that feature. The formula for this is: P(X = k) = (K,k)*(N-K,n-k) / (N,n), which can be simplified to P(X=k) = (K,k)/(N,n), where the notation (A,B) is the binomial coefficient, which stands for the number of ways that we can draw B draws from a population of A without regard to the order, which is A!/(B!(B-a)!).

## RESULTS

**Fig. 1:**
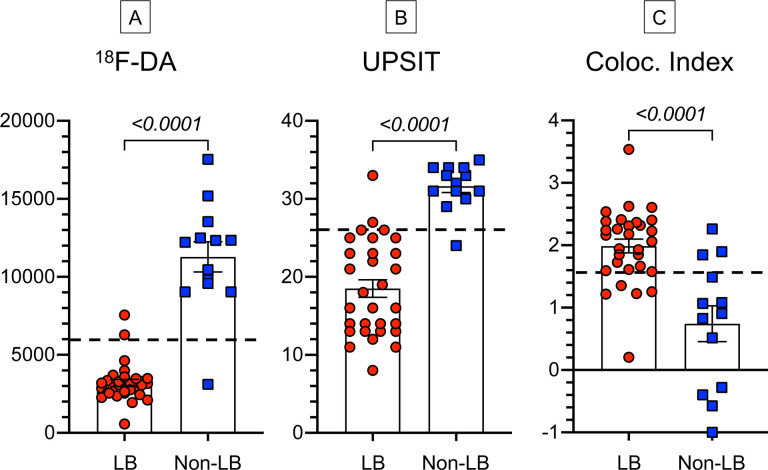
Individual values for (A) ^18^F-dopamine- (^18^F-DA)-derived radioactivity, (B) scores on the University of Pennsylvania Smell Identification Test (UPSIT), and (C) α-synuclein-tyrosine hydroxylase colocalization (Coloc.) indexes in groups with Lewy body (LB, red circles) and non-Lewy body (Non-LB, blue squares) forms of neurogenic orthostatic hypotension. Mean ± SEM values are displayed. Numbers in italics are p values for independent-means t tests comparing the LB vs. non-LB groups. Dashed lines show cutoff values for ^18^F-dopamine-derived radioactivity (normal >6,000 nCi-kg/cc-mCi), UPSIT scores (normal >28), and colocalization indexes (normal<1.57). All 3 biomarkers distinguished the LB from the Non-LB groups, but with overlaps in the data distributions.

The dataset for this study corresponded to evaluations of 44 patients who had ^18^F-dopamine PET, UPSIT scores, and skin biopsies analyzed for α-syn-TH colocalization indexes. In patients with biomarker values over multiple visits, for statistical analyses the values were averaged across the visits. All 3 biomarkers separated the LB from the non-LB forms of nOH (Fig. 1), although there were overlaps in the distributions.

### Trends over years

**Fig. 2:**
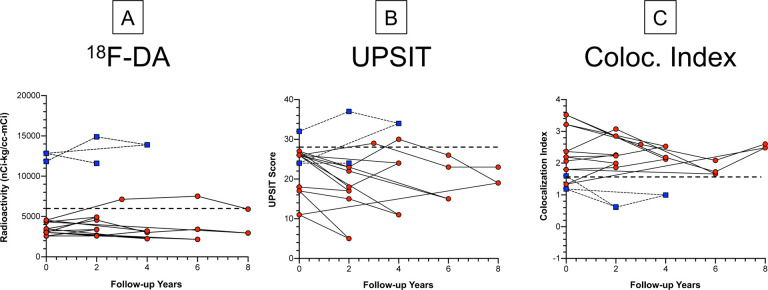
Individual values for (A) ^18^F-dopamine- (^18^F-DA)-derived radioactivity, (B) scores on the University of Pennsylvania Smell Identification Test (UPSIT), and (C) α-synuclein-tyrosine hydroxylase colocalization (Coloc.) indexes as a function of years of follow-up in patients with Lewy body (LB, red circles, solid lines) or non-Lewy body (Non-LB, blue squares, thin dashed lines) forms of neurogenic orthostatic hypotension. Thick dashed lines show cutoff values for ^18^F-dopamine-derived radioactivity (normal >6,000 nCi-kg/cc-mCi), UPSIT scores (normal >28), and colocalization indexes (normal<1.57). For all 3 biomarkers, abnormal values in the LB nOH patients and normal values in the LB No OH patients persisted during follow-up.

For all 3 biomarkers, abnormal values in the subgroup of LB nOH patients with follow-up data persisted over years (Fig. 2); normal values in the small subgroup of non-LB nOH patients also persisted. In both subgroups there were no trends over years in values for any of the biomarkers.

### Biomarker combinations

**Fig. 3:**
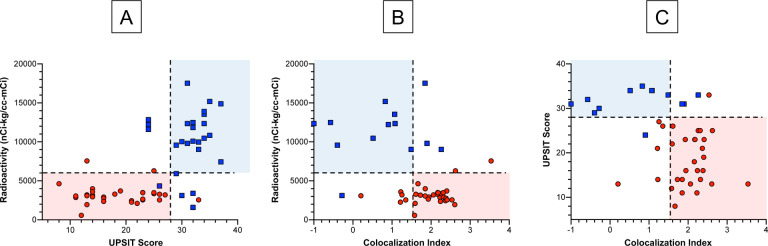
Combinations of 2 biomarkers: (A) UPSIT scores and cardiac ^18^F-dopamine-derived radioactivity, (B) cardiac ^18^F-dopamine-derived radioactivity and α-synuclein-tyrosine hydroxylase colocalization indexes, and (C) UPSIT scores and α-synuclein-tyrosine hydroxylase colocalization indices in groups with Lewy body (LB, red circles) or non-Lewy body (Non-LB, blue squares) forms of neurogenic orthostatic hypotension. Dashed lines show cutoff values for ^18^F-dopamine-derived radioactivity (normal >6,000 nCi-kg/cc-mCi), UPSIT scores (normal >28), and colocalization indexes (normal<1.57). For all 3 biomarker combinations there were distinct but imperfect separations between the LB and non-LB groups, as indicated by the pink and blue rectangles.

Combining cardiac ^18^F-dopamine-derived radioactivity with UPSIT scores efficiently separated the LB from the non-LB patients (Fig. 3A). Among 31 LB nOH patients with data about ^18^F-dopamine-derived radioactivity and UPSIT scores, 28 (90%) had abnormal values for both biomarkers. In contrast, none of 13 non-LB nOH patients (0%) had abnormal values for both biomarkers (p<0.0001 by Fisher’s exact test). For separating the LB and non-LB nOH groups the sensitivity of this biomarker combination therefore was 90% at a specificity of 100%. When cardiac ^18^F-dopamine-derived radioactivity was expressed vs. a-syn-TH colocalization indexes, there was also good separation of the LB nOH from the non-LB nOH group (Fig. 3B). Among 31 patients in the LB nOH group, 24 (77%) had abnormal values for both biomarkers, while 0 of 13 (0%) in the non-LB nOH group had this combination (p<0.0001, sensitivity 77%, specificity 100%).

When UPSIT scores were related to α-syn-TH colocalization indexes, again there was good separation of the LB and non-LB nOH groups (Fig. 3C). Among the 31 patients in the LB group, 24 (77%) had abnormal values for both biomarkers, while 0 of 13 (0%) in the non-LB group had this combination, (p<0.0001, sensitivity 77%, specificity 100%).

**Fig. 4:**
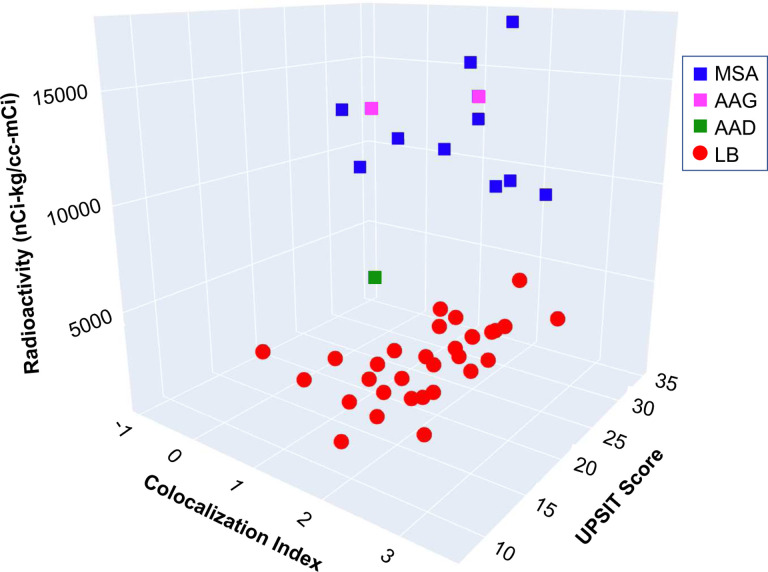
3-D scatter plot showing complete separation of Lewy body (LB, red circles) from non-LB forms of neurogenic orthostatic hypotension, based on cardiac ^18^F-dopamine-derived radioactivity, scores on the University of Pennsylvania Smell Identification Test (UPSIT), and α-synuclein-tyrosine hydroxylase colocalization indexes. Blue squares correspond to patients with multiple system atrophy (MSA), magenta squares patients with autoimmune autonomic ganglionopathy (AAG), and green square a patient with autoimmunity-associated autonomic failure with sympathetic denervation (AAD).

For none of the 2-biomarker combinations was the separation perfect between the LB nOH and non-LB nOH groups. When all 3 parameters were considered together, however, there was complete separation of the LB and non-LB groups (See the 3-D scatterplot in Fig. 4).

### Phosphorylated α-synuclein

**Fig. 5:**
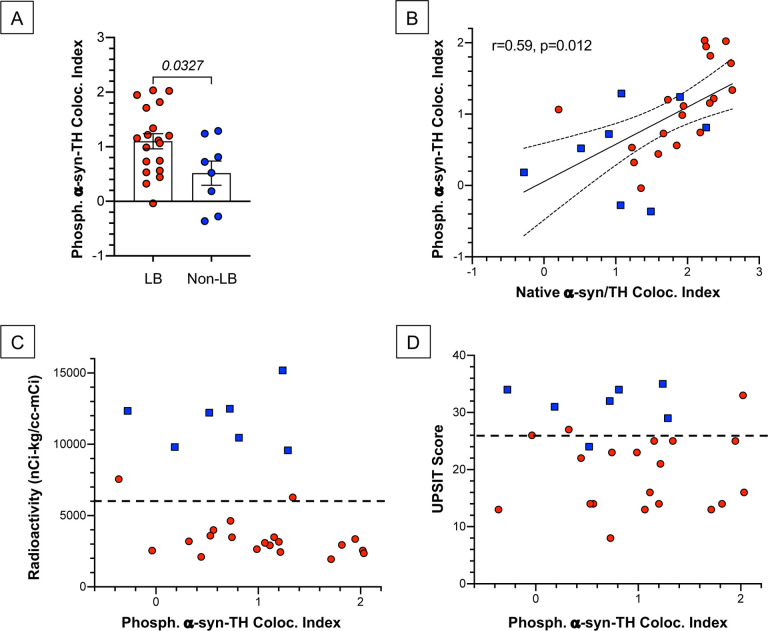
Phosphorylated α-synuclein (α-syn)-tyrosine hydroxylase (TH) colocalization indexes in Lewy body (LB, red) and non-Lewy body (Non-LB, blue) forms of neurogenic orthostatic hypotension (nOH). **(A)** Individual values for phosphorylated α-syn-TH colocalization indexes, with means and SEMs. Number in italics is the p value for the independent-means t-test comparing the LB vs. Non-LB groups. (B) Scatterplot of individual values for phosphorylated α-syn-TH colocalization indexes vs. native α-syn/TH colocalization indexes. The linear regression line of best fit across all subjects is shown with 95% confidence intervals. Also displayed are the Pearson correlation coefficient and p value. (C) Individual values for ^18^F-dopamine-derived radioactivity vs. phosphorylated α-syn-TH colocalization indexes. The dashed line shows the radioactivity cutoff value. (D) Individual values for scores on the University of Pennsylvania Smell Identification Test (UPSIT) vs. phosphorylated α-syn-TH colocalization indexes. The dashed line shows the UPSIT cutoff value.

For distinguishing the LB from the non-LB nOH groups the data for S129 phosphorylated α-syn-TH colocalization indexes were less robust than for native α-syn-TH colocalization indexes (Fig. 5A). Across 27 pairs of data for phosphorylated vs. native α-syn-TH colocalization indexes, the Pearson correlation coefficient was 0.59 (p=0.012) (Fig. 5B). Individual values for phosphorylated α-syn-TH colocalization indexes were unrelated to ^18^F-dopamine-derived radioactivity (Fig. 5C) or to UPSIT scores (Fig. 5D).

### Cluster analysis

**Fig. 6:**
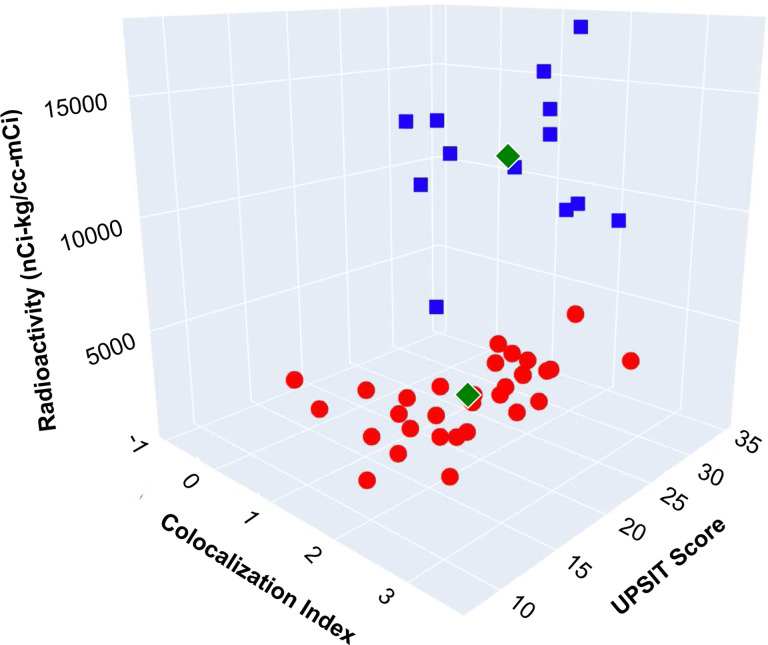
Cluster analysis of data for cardiac ^18^F-dopamine-derived radioactivity, scores on the University of Pennsylvania Smell Identification Test (UPSIT), and α-synuclein-tyrosine hydroxylase colocalization indexes without regard to clinical diagnosis in patients with neurogenic orthostatic hypotension (nOH). The green diamonds show the centroids of the 2 clusters. The data were divided into 2 clusters, with each cluster colored differently (cluster 1=red circles, cluster 2=blue squares). Comparison with Fig. 4 demonstrates that all the data in cluster 1 correspond to patients with LB forms of nOH. The Python code used for the cluster analysis is in Supplementary Table 1.

The hypothesis underlying the cluster analysis was that the combined data for the 3 biomarkers form 2 distinct clusters. Supplementary Table 1 shows the cluster values, and the green diamonds in the 3-D scatterplot in Fig. 6 show the centroids for the 2 clusters. To evaluate the probability of the observed clustering occurring by chance, K = k = n = 31 patients in group 1, and N = 44. P(X = 31) = (31,31)*(44–31,0)/(44,31). Since (31,31) and (13,0) simplify to 1, P(X=31) = 1/(44,31), the value of 1/(44,31) was approximately 1.9 * 10^−11^, indicating indicating an extremely small likelihood that the observed clustering occurred by chance.

All the data in cluster 1 corresponded to patients diagnosed with a LB form of nOH, as demonstrated by the 3-D scatterplots in Supplementary Table 1 and comparison of Fig. 6 with Fig. 4.

## DISCUSSION

In this study we obtained evidence that LB forms of nOH feature low myocardial ^18^F-dopamine-derived radioactivity, low UPSIT scores, and elevated ɑ-syn-TH colocalization indexes in skin biopsies compared to non-LB forms of nOH. All 3 biomarkers separated the groups, although the separations were imperfect. Combining the 3 biomarkers completely distinguished the groups. Conversely, cluster analysis performed on the biomarkers data independently of the clinical diagnosis identified 2 distinct clusters, with 1 of the clusters corresponding exactly to the group with a LB form of nOH.

The abnormalities of cardiac sympathetic innervation, olfaction, and ɑ-syn deposition in sympathetic noradrenergic nerves persisted during follow-up in subgroups of LB nOH and non-LB nOH patients, without upward or downward trends. These findings suggest that the observed biomarker abnormalities were enduring traits. The study was not designed to ascertain when the abnormalities began with respect to the onset of nOH. There are no published studies directly on this key topic. It has been noted that rapid eye movement behavior disorder (RBD), which entails a high risk of development of a central synucleinopathy [52], is associated with OH [51, 53], abnormal cardiac sympathetic neuroimaging [37, 38], olfactory dysfunction [10, 54], and deposition of S129 phosphorylated ɑ-syn in sympathetic noradrenergically innervated skin constituents [1, 5]; however, the timing of onset of these abnormalities with respect to RBD is poorly understood.

Because the UPSIT is effective, simple, inexpensive, widely available, and safe, we think this test (or an analogous test in non-English speakers) should be done in all patients with nOH. In essence this is an objective, quantifiable assessment of the first cranial nerve. A previous publication noted that olfactory dysfunction in PAF was not worse than that in MSA [55], but other studies have found that odor identification is impaired in PAF and not in MSA [12, 27]. In dementia, anosmia is found in DLB and not in Alzheimer’s disease [45] and is common in Alzheimer’s disease that is combined with LB pathology [50]. In patients with RBD the occurrence of olfactory dysfunction predicts early transition to a central LB disease [44].

The present results support our previous findings associating a-syn-TH colocalization with both sporadic and familial LB diseases [31, 32, 34, 42]. Beginning with the publication by Dabby et al. in 2006 [3] most reports on a-syn deposition in skin biopsies from patients with synucleinopathies have been based on immunostaining for the pan-axonal marker protein gene product (PGP) 9.5 [7, 46, 48, 58]. PGP 9.5 does not separate sensory from autonomic neurons and among autonomic neurons does not identify sympathetic noradrenergic fibers specifically.

Indeed, PGP 9.5 may not even be completely specific for neuronal elements [2, 13]. For separating LB from non-LB forms of nOH, we found that a-syn-TH colocalization indexes based on an antibody to native a-syn were superior to those based on an antibody to asyn phosphorylated at the 129S position. Although the LB nOH group had a higher mean colocalization index based on 129S phosphorylated a-syn, and individual values for colocalization indexes by the 2 assay methods were positively correlated, the a-syn-TH colocalization indexes based on 129S phosphorylated a-syn were unrelated to cardiac ^18^Fdopamine-derived radioactivity and to UPSIT scores, whereas indexes based on native a-syn were correlated with values for both biomarkers. It is possible that the antibody used for 129S phosphorylated a-syn cross-reacted with other proteins. Alternatively, since the colocalization method measures the extent of pixel-by-pixel correlations between a-syn and TH signals, if 129S phosphorylated a-syn were present outside catecholaminergic nerve fibers to an appreciable extent, a-syn-TH colocalization indexes could be normal.

The present results confirm that ^18^F-dopamine PET can identify LB forms of nOH [19, 21, 43]. Moreover, a recent prospective, longitudinal study showed that cardiac noradrenergic deficiency revealed by ^18^F-dopamine PET identifies preclinical central LB diseases in at-risk individuals [25].

**Fig. 7:**
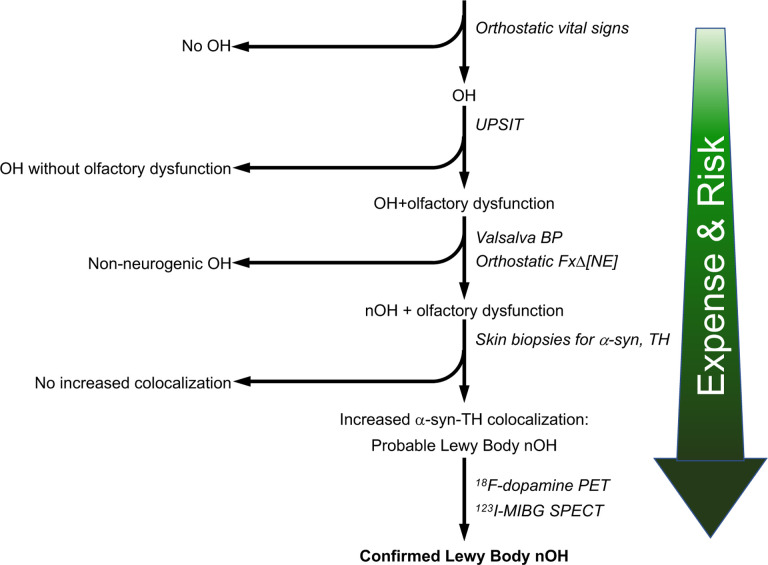
Clinical laboratory algorithm for identifying Lewy body forms of neurogenic orthostatic hypotension (nOH) Each step entails greater diagnostic accuracy but with greater expense, risk, and practical limitations. Olfactory function is assessed by the University of Pennsylvania Smell Identification Test (UPSIT). OH is determined to be neurogenic based on the blood pressure pattern associated with the Valsalva maneuver (Valsalva BP) or on the orthostatic fractional increase in plasma norepinephrine (Orthostatic FxΔ[NE]). Skin biopsies are analyzed to calculate the alpha-synuclein (α-syn)-tyrosine hydroxylase (TH) colocalization index. Other abbreviations: ^123^I-MIBG=^123^I-metaiodobenzylguanidine; PET=positron emission tomography; SPECT=single photon emission computed tomography.

The results of this study seem sufficiently robust for us to propose extending on our previously published algorithm for clinical laboratory evaluation of nOH [28]. In the 4-step algorithm depicted in Fig. 7, olfactory function is assessed by the UPSIT. OH is determined to be neurogenic based on the BP pattern associated with the Valsalva maneuver or the orthostatic fractional increase in plasma norepinephrine. (It should be noted that the ΔHR/ΔBP ratio during tilt table testing is specific but insensitive for detecting nOH [17, 49]). Skin biopsies are assayed for immunoreactive a-syn and TH to calculate the a-syn-TH colocalization index. ^18^F-Dopamine PET is reserved for unusual, difficult differential diagnostic cases or to optimize diagnostic enrichment in experimental therapeutic trials targeting LB forms of nOH. Each sequential step in Fig. 7 entails greater diagnostic accuracy at the costs of greater expense, risk, and practical limitations.

The biomarker triad of cardiac noradrenergic deficiency, olfactory dysfunction, and increased α-syn-TH colocalization indexes separated nOH into 2 distinct clusters, independently of the clinical diagnostic assignment. The p value for such clustering occurring by chance alone was about 2 × 10^−11^, which is to say very close to zero. Importantly, 1 of the 2 clusters corresponded exactly to LB nOH. The present biomarkers data therefore support a phenotypic classification of nOH (LB vs. Non-LB nOH), which could prove valuable for diagnostic enrichment in experimental therapeutic trials.

### Limitations

^18^F-Dopamine PET is only available at the NIH Clinical Center. We hope that the present results will induce other institutions to apply for an IND so that this powerful technology is more widely used. Although ^123^I-MIBG SPECT is available at most centers, insurance carriers in the United States do not cover cardiac ^123^I-MIBG scanning in the diagnostic evaluation of LB diseases. We have commented about this deficiency for many years [9, 26, 39]. The generalizability of the present results therefore is unknown.

### Conclusions

The biomarker triad of cardiac noradrenergic deficiency, olfactory dysfunction, and increased a-syn-TH colocalization indexes efficiently separates LB from non-LB forms of nOH. Conversely, independently of the clinical diagnosis, this combination of biomarkers separates distributions of individual data from nOH patients into 2 distinct clusters, with 1 cluster corresponding to LB nOH. The biomarkers data support a 4-step algorithm for a pathophysiological identification of LB nOH.

## Supplementary Material

1
