# Supplementary material for: Intra-neuronal alpha-synuclein deposition is related to cardiac noradrenergic deficiency and olfactory dysfunction in neurogenic orthostatic hypotension
Source: Res Sq. 2024 Mar 1:rs.3.rs-3988235. Preprint. [Version 1] doi: 10.21203/rs.3.rs-3988235/v1 (PMC10962745; doi:10.21203/rs.3.rs-3988235/v1)
Supplement: 1 [file NIHPPrs3988235V1-supplement-1.pdf]

DATA DICTIONARY FOR SUPPLEMENTARY DATA WORKBOOK

Tab "Neck Ave. if >1 Row"

Cells highlighted in blue with bold text indicate patients with more than 1 dataset; mean data across visits are displayed.

N=subject number

Skin Bx ID #=skin biopsy identification number

AGE=patient age, in years (no data if more than 1 dataset)

SEX=patient sex

GROUP=diagnostic group (PAF=pure autonomic failure; PD+OH=Parkinson's disease with orthostatic hypotension; GBA=glucocerebrosidase mutation heterozygote; MSA=multiple system atrophy; MSA-P=parkinsonian form of multiple system atrophy; AAG=autoimmune autonomic ganglionopathy; AAD=autoimmunity-associated autonomic failure with sympathetic denervation)

GROUP FOR LB nOH ms=patient group, stratified in terms of Lewy body nOH or non-Lewy body nOH

Biopsy Site=body site of skin biopsy (nape of neck)

Colocalization Index=alpha-synuclein/tyrosine hydroxylase colocalization index

Phosphorylated Colocalization Index=phosphorylated alpha-synuclein/tyrosine hydroxylase colocalization index

UPSIT Score=University of Pennsylvania Smell Identification Test score (maximum=40)

18F-DA=^{18}F-dopamine-derived radioactivity in the interventricular septum (8' frame)

UPSIT Abnl. =abnormally low score on the University of Pennsylvania Smell Identification Test score (cutoff=28, abnormal=1, not abnormal=0)

Coloc. Abnl. =abnormally elevated alpha-synuclein/tyrosine hydroxylase colocalization index (cutoff=1.57, abnormal=1, not abnormal=0)

18FDA Abnl=abnormally low ^{18}F-dopamine-derived radioactivity (cutoff=6,000 nCi-kg/cc-mCi, abnormal=1, not abnormal=0)

UPSIT & Coloc. Abnl. =both UPSIT and alpha-synuclein/tyrosine hydroxylase colocalization index abnormal (1) or not both abnormal (0)

UPSIT & 18FDA Abnl. =both UPSIT and ^{18}F-dopamine-derived radioactivity abnormal (1) or not both abnormal (0)

18FDA & Coloc. Abnl. =both ^{18}F-dopamine-derived radioactivity and alpha-synuclein/tyrosine hydroxylase colocalization index abnormal (1) or not both abnormal (0)

All 3 Abnormal=^{18}F-dopamine-derived radioactivity, UPSIT score, and alpha-synuclein/tyrosine hydroxylase colocalization index all abnormal (1) or not all abnormal (0)

Tab "Neck Repeats ≥ 2 Years"

AGE=patient age, in years

SEX=patient sex

GROUP=diagnostic group (PAF=pure autonomic failure; PD+OH=Parkinson's disease with orthostatic hypotension; GBA=glucocerebrosidase mutation heterozygote; MSA=multiple system atrophy; MSA-P=parkinsonian form of multiple system atrophy; AAG=autoimmune autonomic ganglionopathy; AAD=autoimmunity-associated autonomic failure with sympathetic denervation)

GROUP FOR LB nOH ms=patient group, stratified in terms of Lewy body nOH or non-Lewy body nOH

Biopsy Site=body site of skin biopsy (nape of neck)

F/U Years=years since initial evaluation

Colocalization Index=alpha-synuclein/tyrosine hydroxylase colocalization index

UPSIT Score=University of Pennsylvania Smell Identification Test score (maximum=40)

18F-DA=^{18}F-dopamine-derived radioactivity in the interventricular septum (8' frame)

Supplementary Table 1: Python code and data for the 3-D scatterplots in Figs. 4 and 6.

```
import pandas as pd
from io import StringIO

import pandas as pd
import numpy as np
from sklearn.cluster import KMeans
import plotly.graph_objects as go
import plotly.express as px


# Data as a multi-line string
data_string = """
GROUP,Colocalization Index,UPSIT Score,Radioactivity (nCi-kg/cc-mCi),
LB nOH,2.4011,19,3699,
LB nOH,2.2273,11,2862,
LB nOH,1.7261,14,3157,
LB nOH,2.6068,13,1943,
LB nOH,2.3178,14,2943,
LB nOH,2.0617,13,3193,
LB nOH,1.9440,16,2913,
LB nOH,2.1715,23,3496,
LB nOH,2.1602,18,3294,
LB nOH,2.3661,21,2445,
LB nOH,1.5922,22,2102,
LB nOH,2.5353,33,2553,
LB nOH,0.2041,13,3085,
LB nOH,1.2157,21,2267,
LB nOH,2.3832,16,2759,
LB nOH,1.6103,26,3282,
LB nOH,1.5776,12,570,
LB nOH,1.8484,11,3055,
LB nOH,1.8494,14,3985,
LB nOH,2.4101,23,2549,
LB nOH,2.6239,25,6278,
LB nOH,2.2391,16,2354,
LB nOH,1.3525,26,2547,
LB nOH,2.2595,25,3356,
LB nOH,1.2251,14,3587,
LB nOH,1.2547,27,3194,
LB nOH,1.9276,23,2647,
LB nOH,2.3106,25,3481,
LB nOH,1.6652,8,4627,
LB nOH,2.1778,23,3476,
LB nOH,3.5364,13,7555,
```

```
Non-LB nOH,1.4900,33,9036,
Non-LB nOH,-0.5740,32,12489,
Non-LB nOH,0.9047,24,12215,
Non-LB nOH,0.5151,34,10462,
Non-LB nOH,2.2609,33,9036,
Non-LB nOH,0.8271,35,15182,
Non-LB nOH,1.8959,31,9810,
Non-LB nOH,-0.2795,30,3108,
Non-LB nOH,-0.3979,29,9581,
Non-LB nOH,1.0792,34,12341,
Non-LB nOH,1.0678,34,13539,
Non-LB nOH,1.8457,31,17527,
Non-LB nOH,-1.0000,31,12346,
"""
# Using StringIO to simulate a file object
data_io = StringIO(data_string)

# Create a DataFrame, using the first line as headers and ignore the trailing commas
df = pd.read_csv(data_io, sep=",")

# Display the DataFrame
df.head()
# Create a figure object
fig = go.Figure()

# Add LB nOH group in red circles
fig.add_trace(go.Scatter3d(
    x=df[df['GROUP'] == 'LB nOH']['Colocalization Index'],
    y=df[df['GROUP'] == 'LB nOH']['UPSIT Score'],
    z=df[df['GROUP'] == 'LB nOH']['Radioactivity (nCi-kg/cc-mCi)'],
    mode='markers',
    marker=dict(color='red', size=5, symbol='circle'),
    name='LB nOH',
    text=df[df['GROUP'] == 'LB nOH']['GROUP'],
    hoverinfo='text+x+y+z'
))

# Add non-LB nOH group in blue squares
fig.add_trace(go.Scatter3d(
    x=df[df['GROUP'] == 'Non-LB nOH']['Colocalization Index'],
    y=df[df['GROUP'] == 'Non-LB nOH']['UPSIT Score'],
    z=df[df['GROUP'] == 'Non-LB nOH']['Radioactivity (nCi-kg/cc-mCi)'],
    mode='markers',
    marker=dict(color='blue', size=5, symbol='square'),
    name='Non-LB nOH',
    text=df[df['GROUP'] == 'Non-LB nOH']['GROUP'],
```

```
    hoverinfo='text+x+y+z'
))

font_size = 10
# Update the layout to include the custom font settings
fig.update_layout(
    scene=dict(
        xaxis_title='Colocalization Index',
        yaxis_title='UPSIT Score',
        zaxis_title='Radioactivity (nCi-kg/cc-mCi)',
        xaxis=dict(title_font=dict(family="Arial", size=font_size, color="black"),
                   tickfont=dict(family="Arial", size=font_size)),
        yaxis=dict(title_font=dict(family="Arial", size=font_size, color="black"),
                   tickfont=dict(family="Arial", size=font_size)),
        zaxis=dict(title_font=dict(family="Arial", size=font_size, color="black"),
                   tickfont=dict(family="Arial", size=font_size)),
    ),
    font=dict(family="Arial", size=font_size),
    width=800,  # Set the width of the plot
    height=800,  # Set the height of the plot
)

# Show the figure
fig.show()

from sklearn.preprocessing import StandardScaler
from sklearn.cluster import KMeans

# Extract the relevant features
X = df[['Colocalization Index', 'UPSIT Score', 'Radioactivity (nCi-kg/cc-mCi)']].values

# Normalize the features
scaler = StandardScaler()
X_normalized = scaler.fit_transform(X)

# Perform K-Means clustering on the normalized data
kmeans = KMeans(n_clusters=2, random_state=0, n_init=10).fit(X_normalized)

# Add the cluster labels to the original DataFrame
df['Cluster'] = kmeans.labels_

# Inverse transform the centroids to original scale
centroids_original_scale = scaler.inverse_transform(kmeans.cluster_centers_)

# Plot the data and the centroids
fig = go.Figure()
```

```
# Add the data points for cluster 0
fig.add_trace(go.Scatter3d(
    x=df[df['Cluster'] == 0]['Colocalization Index'],
    y=df[df['Cluster'] == 0]['UPSIT Score'],
    z=df[df['Cluster'] == 0]['Radioactivity (nCi-kg/cc-mCi)'],
    mode='markers',
    marker=dict(size=5, color='red',symbol='circle'),
    name='Cluster 0'
))

# Add the data points for cluster 1
fig.add_trace(go.Scatter3d(
    x=df[df['Cluster'] == 1]['Colocalization Index'],
    y=df[df['Cluster'] == 1]['UPSIT Score'],
    z=df[df['Cluster'] == 1]['Radioactivity (nCi-kg/cc-mCi)'],
    mode='markers',
    marker=dict(size=6, color='red',symbol='circle'),
    name='Cluster 1'
))

# Add the centroids (transformed back to the original scale)
fig.add_trace(go.Scatter3d(
    x=centroids_original_scale[:, 0],
    y=centroids_original_scale[:, 1],
    z=centroids_original_scale[:, 2],
    mode='markers',
    marker=dict(size=10, color='green', symbol='diamond'),
    name='Centroids'
))

# Update the layout for a clearer view
fig.update_layout(
    scene=dict(
        xaxis_title='Colocalization Index',
        yaxis_title='UPSIT Score',
        zaxis_title='Radioactivity (nCi-kg/cc-mCi)'
    ),
    width=800,  # Set the width of the plot
    height=800,  # Set the height of the plot
)

# Show the figure
fig.show()
```
